# Traumatic Optic Neuropathy Is Associated with Visual Impairment, Neurodegeneration, and Endoplasmic Reticulum Stress in Adolescent Mice

**DOI:** 10.3390/cells10050996

**Published:** 2021-04-23

**Authors:** Shelby M. Hetzer, Fernanda Guilhaume-Correa, Dylan Day, Alicia Bedolla, Nathan K. Evanson

**Affiliations:** 1 Neuroscience Graduate Program, University of Cincinnati College of Medicine, Cincinnati, OH 45267, USA; canslesy@mail.uc.edu (S.M.H.); dylanday1@gmail.com (D.D.); bedollam@mail.uc.edu (A.B.); 2 Translational Biology, Medicine and Health, Virginia Polytechnic Institute and State University, Roanoke, VA 24016, USA; fergc92@vt.edu; 3 Division of Pediatric Rehabilitation Medicine, Cincinnati Children’s Hospital Medical Center, Cincinnati, OH 45229, USA; 4 Department of Pediatrics, University of Cincinnati, Cincinnati, OH 45229, USA

**Keywords:** traumatic optic neuropathy, head trauma, adolescent head trauma, ER stress, mice

## Abstract

Traumatic brain injury (TBI) results in a number of impairments, often including visual symptoms. In some cases, visual impairments after head trauma are mediated by traumatic injury to the optic nerve, termed traumatic optic neuropathy (TON), which has few effective options for treatment. Using a murine closed-head weight-drop model of head trauma, we previously reported in adult mice that there is relatively selective injury to the optic tract and thalamic/brainstem projections of the visual system. In the current study, we performed blunt head trauma on adolescent C57BL/6 mice and investigated visual impairment in the primary visual system, now including the retina and using behavioral and histologic methods at new time points. After injury, mice displayed evidence of decreased optomotor responses illustrated by decreased optokinetic nystagmus. There did not appear to be a significant change in circadian locomotor behavior patterns, although there was an overall decrease in locomotor behavior in mice with head injury. There was evidence of axonal degeneration of optic nerve fibers with associated retinal ganglion cell death. There was also evidence of astrogliosis and microgliosis in major central targets of optic nerve projections. Further, there was elevated expression of endoplasmic reticulum (ER) stress markers in retinas of injured mice. Visual impairment, histologic markers of gliosis and neurodegeneration, and elevated ER stress marker expression persisted for at least 30 days after injury. The current results extend our previous findings in adult mice into adolescent mice, provide direct evidence of retinal ganglion cell injury after head trauma and suggest that axonal degeneration is associated with elevated ER stress in this model of TON.

## 1. Introduction

Traumatic brain injuries (TBIs) are diverse in cause, severity, location, and duration of symptoms. Although a number of models have been used in research settings, many facets of injury require further exploration including injury to the optic/visual system. Visual deficits are common after TBI and can be due to damage to nearly any part of the brain, including the eye or optic nerve [1]. Severe and moderate deficits seen after TBI consist of complete loss of vision, loss of visual fields, loss of color vision, or a decrease in visual acuity. Further, many symptoms may not be identified immediately, which can lead to a missed diagnosis, especially in connection to the head injury [2]. Visual sequelae of TBI can also cause significant issues with recovery [3] and can impact functional outcomes in children after even mild injuries [4].

Among the pathophysiologic mechanisms of TBI-associated visual deficits is traumatic optic neuropathy (TON), which is particularly associated with facial or frontal head impact and can lead to severe visual impairment [5]. TON is often further classified as either direct (involving direct, penetrating injury) or indirect (injury due to transmission of force through the skull into the optic nerve or due to secondary mechanisms like increased intraocular/intracranial pressure within the optic canal, axonal shearing, or neuroinflammatory responses). Indirect traumatic optic nerve damage, in particular, is a known comorbidity of traumatic head injury in both adolescents and adults, with a reported incidence of between 2% and 5.2% of patients with closed head injury [5,6]; however, it is likely that reported incidence rates are underestimated [7]. What is more, roughly 80% of cases are reported in young adult males, of which 20% occur in children [8].

While a direct optic nerve injury can be diagnosed by detecting optic nerve (ON) avulsion, ON transection, or ON sheath hemorrhage (e.g., by imaging studies), indirect TON is generally more difficult to distinguish [9]. Fractures of the optic canal can be seen on CT if present, but often, injuries can only be detected by vision testing [9] such as optical coherence tomography, which has revealed that indirect TON (iTON) causes significant thinning and deterioration of retinal layers at the time of injury and up to 35 years later [2]. Imaging of iTON within the brains of living human subjects is possible through diffusion tensor imaging and reveals that mild TBI can produce axonal injury in optic radiations [9,10,11] and the anterior thalamic radiations from the lateral geniculate body [10]. However, even advanced imaging modalities are limited in the information they can provide.

Most studies of human and animal optic nerve injury have focused on models relevant to direct traumatic optic neuropathy (e.g., optic nerve crush or transection models), leaving both diagnosis and study of indirect TON less understood [12,13,14]. While these models provide valuable information into direct visual neuropathies, they are less relevant to indirect injury, which may be more common in patients with mild to moderate TBI [9]. A few TBI models have reported optic tract injury along with other diffuse axonal injury [15,16,17], and, although many utilize repetitive TBI models [18,19,20,21], there are now at least three different proposed models employing a single impact [22,23,24]. Moreover, studies on optic nerve injury have only recently begun to analyze central projections of the optic nerve like the lateral geniculate nucleus [16,20,25,26], superior colliculi [20,22], and supra-oculomotor nucleus and caudate [20] but none examine adolescent populations. This is presumably because adult populations, such as military personnel, have higher incidence rates than children. However, as we and others showed, outcomes differ between mice separated by as little as two weeks of age after mild TBI in memory performance, mortality, and severity of brain pathophysiology [25,26,27,28,29,30,31]. This is important, as optic nerve injury has been reported in adolescent-aged mice [32,33] and human children [8], so it is likely that these differences might be present across age groups in the visual system. 

Finally, there are no evidence-based treatment options currently available for improving outcomes after TON [34]. A better understanding of the pathophysiology of TON is likely needed to develop more rational treatment approaches. For example, reactive oxygen species could be a potential target for treatment based on research, showing this as an acute consequence of iTON [24]. Research in other optic neuropathy models also suggests that the unfolded protein response (UPR) may be involved in optic nerve degeneration [35]. For example, the UPR-initiating chaperone immunoglobulin heavy chain-binding protein (BiP) is activated in retinitis pigmentosa [14]. In models of optic nerve crush, CHOP expression is increased, and, with CHOP knockdown, RGCs have increased survival [35]. 

Accordingly, we developed a reproducible murine model of indirect traumatic optic neuropathy associated with head trauma. We have previously reported degeneration in the optic tract, lateral geniculate nucleus, and superior colliculus within one week after TBI in adult male mice, but TON has not yet been studied in an adolescent population [23,25,26,27,28,29,30,31]. Therefore, the goal of the current studies was threefold: (1) to expand our previous findings by including measures of retinal ganglion cell (RGC) loss, changes to visual acuity, and changes in subcortical targets of the optic nerve both one week and one month after injury; (2) to determine the extent of visual impairment and optic system damage in adolescents; (3) and to explore the hypothesis that endoplasmic reticulum stress and reactive oxygen species (i.e., the UPR) may be interconnected mechanisms through which early and lasting damage is propagated through the visual system.

## 2. Materials and Methods

### 2.1. Animals

These experiments were performed in 6-week-old adolescent [36] male C57BL/6J mice (Jackson Laboratories, Bar Harbor, ME, USA). Animals were housed under a 14h:10h light:dark schedule in pressurized individually ventilated cage racks, with 4 mice per cage, and were given ad libitum access to water and standard rodent chow. All animal procedures were performed in accordance with United States animal protection rules, in accordance with The Guide for Animal Care and Use, and approved by the University of Cincinnati Institutional Animal Care and Use Committee (protocol # 17-04-03-01, approved 5 November 2017). Animals habituated to the vivarium for one week prior to undergoing moderate head trauma and subsequent procedures.

### 2.2. Traumatic Brain Injury

**Figure 1:**
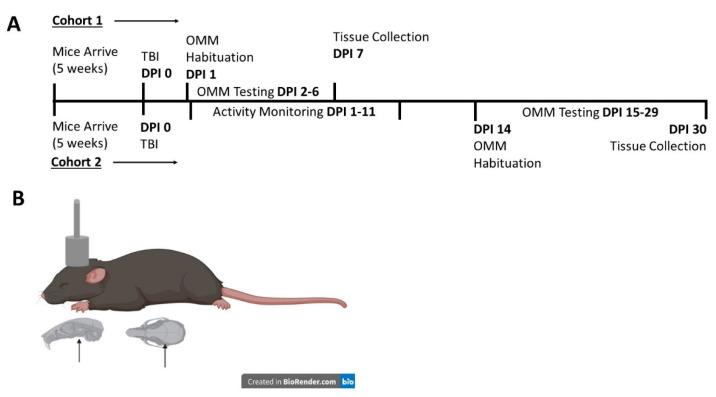
Experimental outline. Two cohorts of mice were utilized in this study. (**A**) shows a timeline for described experiments. (**B**) shows an estimate of our weight drop location with scalp intact. Arrows indicate the location of bregma. OMM = Optomotor Machine. Images in (**B**) were created in BioRender at BioRender.com.

A closed-head TBI injury was performed by weight drop, as previously described [37]. In brief, mice were anesthetized using isoflurane (2–3%) and positioned under a metal rod (1.2 cm diameter; 400 g), raised to 1.5 cm above the scalp in prone position on a 0.5-cm-thick piece of cork board. An injury was produced by dropping the rod onto the calvarium with scalp intact, approximately above the bregma (Figure 1B). Compared to other weight drop methods, this approach uses a greater weight from a lower height and appears to lead to injury to the optic nerve within the optic canal, but generally sparing other areas of the brain [23]. After injury, mice were immediately removed from the apparatus and allowed to recover. During recovery, obvious clinical seizures were observed and noted. Sham animals were subjected to anesthesia, then allowed to recover without undergoing the TBI procedure. Mice were monitored post-TBI for evidence of pain behaviors, and analgesia was available for mice that did not recover quickly; however, analgesia was not required. Cohorts of mice were used for retinal (*n* = 9–12) and brain histology (*n* = 7–10), protein analysis (*n* = 8–17), and behavioral analysis (*n* = 8–12). The number of subjects in histological data varies due to either overlapping tissue or torn tissue after mounting, which prevented accurate measurements, or to significant outliers determined by Grubbs test. The variance in the number of subjects in Western blot data occurred where the amount of protein extracted from some mice was too low to analyze all the markers presented. Figure 1A illustrates the timeline of experimental procedures performed, and Figure 1B depicts the location of the head injury. 

### 2.3. Behavioral Testing

#### 2.3.1. Circadian Rhythm and Locomotion

Twenty-four hours after injury, animals were weighed and separated into individual cages with ad libitum access to food and water but without any enrichment (e.g., no cotton bedding). They were then placed into activity monitors (San Diego Instruments PAS system, *N* = 12, and Lafayette Activity Monitoring System, *n* = 8). The systems were both set to record movements as beam breaks for 24–48-h time blocks. Every 24–48 h, mice were weighed, and the systems were reset. This was repeated for 11 days postinjury. Beam breaks were binned into 1-h periods for analysis. Activity monitors were used to analyze possible circadian rhythm shifts and overall activity levels.

#### 2.3.2. Optomotor/Optokinetic Response (OKR) and Visual Acuity Testing

##### Optomotor Machine (OMM) 

A machine was built to test optomotor reflexes, similar in design to the apparatus previously described [38] and illustrated in Figure 3A. The plexiglass case surrounding the mouse measures 20.2 inches high by 10 inches in diameter. The central pedestal measures 7.4 inches tall by 3 inches wide, leaving roughly 3 inches of space between the mouse and the plexiglass on all sides. Sine-wave gratings are centered to extend 4 inches above and below the pedestal to encompass the mouse’s full frame of vision. The pedestal remains stationary as the plexiglass rotates on a white circular stand. Speeds could be adjusted from 1 revolution per minute (rpm) up to 6 rpm, and the direction of a spin could be switched between clockwise and counterclockwise. 

##### Sine-Wave Gratings

Sine-wave visual gratings were generated using the python package Psychopy [39] at varying spatial frequencies (i.e., contrasts): 0.12, 0.26, 0.32, 0.39, and 0.42 cycles per degree (cpd) depicted as vertical, alternating, black and white lines. Each grating could be interchanged and attached to the inside of the machine (Figure 3B). A series of 15 gratings ranging from 0.03–0.64 cpd were piloted (data not shown) based on the reported ranges for mouse visual acuity, the aforementioned five were chosen based on the optimal performance (i.e., consistently present optokinetic response) in normal C57BL/6J mice [38,40]. A HOBO luminance detector (Part#H08-004-02; Onset Computer Corporation, Bourne, MA, USA) was used to determine the room illumination. As per IACUC’s recommended illumination, the animal behavior room’s luminance was 380 Lux with the lights on. Gratings were printed on regular printer paper and did not change the luminance inside the machine.

##### Testing

Order of subject, grating spin direction (i.e., clockwise or counterclockwise), and grating frequency were randomized across 5 days using a random number generator (as a result, there were no effects of animal order (or time tested), day of testing, or spin data not shown). Animals were placed on a pedestal in the optomotor machine without a grating and were allowed to habituate for 10 min on day 1 (either 24 h after injury for the 7 days postinjury (DPI) cohort or 15 DPI for the 30 DPI cohort); after 5 min, the machine was turned on and rotated. On day two of testing, animals were placed on the raised platform with the grating already in place (to avoid unnecessary disturbances and to prevent reactions to events that could be seen through the plexiglass cylinder) and were allowed to acclimate to the device for 5 min (with no rotation). The machine was then turned on and rotated in the first direction for 2 min at 2 rpm. The machine was then stopped, and a rest period of 30 s elapsed before it was turned on again for 2 min in the reverse direction (protocol adapted from [41]). This procedure was repeated 5 days in a row at the same time each day by the same experimenter (between 0830-1430, with each mouse taking about 10 min plus cleaning and resetting the equipment). One mouse was tested at a time, with only one grating tested per day. The machine was cleaned using an antiseptic wipe between each animal. A video camera was secured on a ceiling mount 2.5 feet above the device and recorded the last minute of acclimation and the 4.5 min of the testing time. A screen capture of the recording area is provided in Supplementary Figure S1.

##### Scoring

OKRs were tallied for the same amount of recording time for each animal (i.e., 4 min of rotation time) from video footage collected on a SONY HDR-CX440 Handycam. Each video was viewed and scored by two trained reviewers who were blinded to experimental conditions. Reviewers were trained for at least 2 to 3 weeks on optomotor response criteria before scoring videos, and there were no significant inter-rater differences. Briefly, an optokinetic response was defined as a rotation of the nose consistent with the speed and direction of the drum over >5° angle changes. The total number of OKRs for each grating were recorded, and the average between the two reviewers was analyzed.

### 2.4. Retina Dissection

For both cohorts, animals were euthanized and eyes removed at 7 or 30 days after TBI. After a fatal overdose of pentobarbital (Fatal Plus^®^), the **left** eye was proptosed, enucleated, and placed in ice-cold 1× PBS. The retina was immediately dissected on ice within 10 min using an adapted protocol from Ullmann et al. (2012) [42]. In a petri dish with 1× PBS, the eye was punctured just above the ora serrata, and microdissection scissors were used to cut along the cornea and iris until the corneal surface was removed. The lens, iris, and vitreous humor were then removed without touching the retinal surface of the interior eyecup. Finally, the sclera was gently pulled away from the retina, and the retina was separated intact for protein extraction. The left retina was then immersed in lysis buffer (20-mM Tris-HCL, pH 7.4, 2-mM EDTA, 0.5-mM EGTA, 1-mM DTT, and HALT protease/phosphatase inhibitor) and flash-frozen on dry ice. Retinas were then stored at −80 °C until protein extraction (as described below). 

For cohort 1 (7 DPI), the right eye was removed after perfusion of the animal with 4% PFA by proptosing the eye and using curved scissors to sever the optic nerve for immunohistochemistry (IHC). The enucleated whole right eye was post-fixed in ice-cold 4% PFA for 1 h, then submerged in 30% sucrose overnight. Eyes remained in sucrose at 4 °C until ready for retina removal, as described above. For cohort 2 (30 DPI), our protocol was adjusted slightly (due to us learning how to do this more smoothly), and both eyes were removed before perfusion rather than after. The same subsequent procedure for post-fixation of the right eye for IHC was followed, as in cohort 1; this did not alter the appearance of tissues or cell counts, as can be seen between the sham conditions for 7 versus 30-day tissue in Figures 5 and 6B,E,F. Additionally, there were no comparisons made between the cohort 1 and cohort 2 samples. Once ready for IHC (for both cohorts), the right eye was washed three times for five minutes in 1× PBS, stored at 4 °C in 1× PBS in 1.7-mL centrifuge tubes, and stained within 48 h of dissection (see Section 2.6 below for the IHC protocol). 

### 2.5. Western Blots

To extract retinal protein, frozen left retinas in lysis buffer were thawed and homogenized with a pellet homogenizer for roughly 10 s. Samples were sonicated in a cold water bath for 5 min; after which, they were centrifuged at 3000 rpm for 20 min. Supernatant was removed, and a BCA protein assay (Pierce BCA Protein Assay Kit; Thermo Fisher Scientific, Waltham, MA, USA; cat# 23227) was used to calculate the protein concentration. Twenty microgram samples were prepared for Western blotting by adding β-mercaptoethanol and NuPAGE LDS 4× sample buffer (Invitrogen, Waltham, MA, USA; cat# NP0007) and heating at 70 °C for 10 min. Using SurePAGE, 4–12% bis-tris gels (GenScript, Piscataway, NJ, USA; cat# M00653) and 40 μL of protein sample was loaded into each well and run at 200V for roughly 1.5 h in Tris-MOPS-SDS running buffer (GenScript; cat# M00138). Gels were transferred via a wet transfer using Transfer Buffer Powder in 1× TBST (GenScript; cat# M00139) for 1 h at 30V onto Amersham Hybond P 0.45 PVDF membranes (GE Life Sciences, Pittsburgh, PA, USA; cat# GE 10600029). After transfer, membranes were rinsed in TBST and blocked for one hour in either 5% milk (PERK, Caspase-3, and ERO1L) or 5% BSA (βActin, IRE-1α, CHOP, and PDI). 

Membranes were then incubated 24–48 h at 4 °C in primary antibodies PERK (1:500 anti-Rbt; Cell Signaling Technologies (CST), Danvers, MA, USA; cat# 3192S), IRE-1α (1:500 anti-Rbt; CST; cat# 3294S), PDI (1:2000 anti-Rbt; CST; cat# 3501S), CHOP (1:500 anti-ms; CST; cat# 2895S), caspase-3 (1:5000 anti-Rbt; CST; cat# 9662S), ERO1-L (1:1000 anti-Rbt; Thermo Fisher Scientific; cat# 702709), and β-Actin (1:3000 anti-ms; CST; cat# 3700S). After incubation, membranes were rinsed in TBST, then incubated in respective anti-mouse HRP (CST; cat# 7076S) or anti-rabbit HRP (CST; cat# 7074S) for 1 h at room temperature. Finally, membranes were washed and incubated in Pierce ECL PLUS Western blotting substrate (Fisher Scientific; cat# 32132) for 1–5 min; after which, they were placed between transparency film and taken to a dark room for exposure (times ranged from 2 min to 4 h). Films (CL-XPosure Film; Thermo Scientific; cat# PI34090) were scanned and converted to 8-bit TIFs for analysis using ImageJ software [43]. Using the Gel Analysis tool peaks across protein bands were recorded. Each protein band was normalized to β-Actin.

### 2.6. Histology

For a histologic analysis of the brain tissue, mice were euthanized using pentobarbital (Fatal Plus^®^) 7 days and 30 postinjury, as previously described [25]. Thirty-five micrometer sections were stained using Fluoro-Jade B or fluorescent immunohistochemistry. Fluoro-Jade B (Histo-Chem, Jackson, AR, USA; cat# 1FJB), a marker for degenerating neurons and axons [44], was used according to the manufacturer’s directions. After staining, slides were allowed to air dry completely in the dark and left un-coverslipped to avoid a high background (Electron Microscopy Sciences, Hatfield, PA, USA; cat# 13512). Slides were stored in a slide box and kept in the dark until imaging. 

### 2.7. Immunofluorescence

Primary antibodies used for immunofluorescence on brain sections were polyclonal rabbit anti-glial fibrillary acidic protein (GFAP; DAKO, Santa Clara, CA, USA; cat # Z0334; RRID AB_10013382) and ionized calcium-binding adaptor molecule 1 (Iba-1; Synaptic systems, Goettingen, Germany; cat# 234003, RRID AB_10641962), both at 1:2000 dilutions. Brain sections were washed in PBS and then incubated in blocking solution (PBS with 0.1% bovine serum albumin and 0.4% Triton X-100) for 1 h. Following this, sections were incubated overnight at 4 °C with primary antibody in blocking solution. On the second day, sections were washed, then incubated with Cy-3 conjugated secondary antibody (Jackson Immunochemicals, West Grove, PA, USA; cat# 711-165-152, RRID AB_2307443) at 1:500 dilution for 1 h and covered at room temperature. Sections were mounted in PBS with 1 mL of 5% gelatin added, allowed to dry in the dark, rinsed in water, and allowed to dry again. Slides were coverslipped using antifading polyvinyl alcohol mounting medium (Sigma-Aldrich, St. Louis, MO, USA). 

Primary antibodies used for immunofluorescence on whole-mounted retinal sections were brain-specific homeobox/POU domain protein 3A (Brn3a; Millipore, Burlington, MA, USA; cat# MAB1585; RRID:AB_94166) at 1:1000 and GFAP (1:1000). Brn3a-positive cells were confirmed with the inclusion of a secondary only control for which there was no nonspecific binding detected (see Supplementary Figure S2). DAPI staining was achieved with Vectashield Antifade Mounting Medium with DAPI (Vector Laboratories; cat# H-1200; RRID: AB_2336790). All steps were performed in a centrifuge tube to avoid contact and handling of the retina. Retinas were washed in PBS, incubated in 0.3% H_2_O_2_ for 20 min, washed again, then permeabilized in 0.5% Triton X-100 for 15 min at −80 °C. Tissue was thawed, washed in fresh permeabilization solution for 20 min, then blocked (2% TX-100, 2% BSA, and 5% normal goat serum in PBS) for 1 h at room temperature. Retinas were then incubated in Brn3a primary antibody 48–72 h at 4 °C. After primary incubation, retinas were washed in 0.5% TX-100 and incubated for 1.5 h in anti-mouse biotinylated secondary antibody (1:400; Vector Laboratories; cat# BA-9200; RRID: AB_2336171). Washes were performed followed by treatment in VECTASTAIN Elite ABC HRP Kit (1:800; Vector Laboratories; cat# PK-6100; RRID: AB_ 2336817) for 1 h. Following ABC, retinas were incubated in Cy3 streptavidin (1:500; Invitrogen, Grand Island, NY, USA; cat# 434315) for 2 h at room temperature covered. Retinas were then washed, incubated in blocking solution for 30 min, and left in GFAP primary antibody 24–48 h at 4 °C. After incubation in the second primary antibody, retinas were washed, incubated in anti-rabbit Alexa 488 (1:500; Invitrogen; cat# A11034) for 2 h at room temperature, washed, and then mounted. 

Retinas were mounted onto positively charged slides pretreated with Gattenby’s Solution (0.5% gelatin, 0.05% chromium potassium sulfate dodecahydrate (CrK(SO_4_)_2_·12H_2_O; Fisher Scientific, Grand Island, NY, USA; cat# C337-500) in ddH_2_O. To do this, whole retinas were transferred to slides in 1× PBS, and 4-5 radial cuts were made to create a “petal” or “cross” shape, allowing the retina to lie flat on the slide (see Figure 5J). 

### 2.8. Image Analysis

Photomicrographs of all brain slides were taken by a blinded observer, using an Axio lmager Z1 microscope with an Apotome (Leica Microsystems, Buffalo Grove, IL, USA). All slides were photographed using the same exposure time and magnification within the planned comparison groups. FJ-B-stained slides and retinal whole mounts were viewed on a Nikon C2 Plus Confocal Microscope (Nikon Corporation, Melville, NY, USA). FJ-B slides were imaged using the same fluorescence intensity (on the FITC channel) and background reduction within comparison groups and magnifications. Retinal slides were imaged using three channel wavelengths for TRITC-FITC-DAPI, because these images were only used for cell counting, fluorescence, and background reduction were corrected for each image to allow the best representation of cells within each region of interest. Retinas were separated into three zones (peripheral, mid-peripheral, and center), with 3–5 images (depending on how many times a retina was cut) taken of each zone for each retina (Figure 5J). Images within each zone were averaged to get a representative retinal ganglion cell count for that zone; this number was used in the statistical analyses.

Image analysis and quantification were performed using ImageJ software [43] for GFAP and FJ-B images or NIS elements software (Nikon, Melville, NY, USA) for Iba-1 and Brn3a. For GFAP and FJ-B images, the mean fluorescence intensity (MFI) was measured in multiple nonoverlapping samples within the relevant region. The mean fluorescence intensity was chosen for FJ-B due to the non-somatic nature of our staining, which prevented cell counting for quantification. Luminance and microscope settings could be held constant and all tissue within an experimental group stained at the same time, which allowed us to control for confounding variables of MFI measurements. For Iba-1-stained tissue, images were thresholded, and automated soma perimeter measurements were taken for all cells above the threshold within the region of interest. Comparisons were performed only within a single region. Brn3a cell counts were acquired using NIS elements automated detection settings and were thresholded so that only complete, nonoverlapping cells were counted. 

### 2.9. Statistical Analysis

Statistical analysis was performed using the SigmaPlot software package (Systat, San Jose, CA, USA); 3-way comparisons were analyzed using Statistica (TIBCO, Palo Alto, CA, USA) for activity monitoring (treatment × lighting × day). Weight gain (treatment × day) and OKR data (treatment × grating) were analyzed using 2-way analysis of variance (ANOVA) with repeated measures. Significant effects were further analyzed using the Holm-Sidak post-hoc method. Seven days postinjury ventral LGN FJ-B and PERK data were analyzed with nonparametric Mann–Whitney rank sum when the data failed to pass normality. Histologic and protein measures were analyzed using an unpaired *t*-test as the preinjury weight × injury, preinjury weight × survival, and seizure × righting time analyses. Preinjury weight and righting time were also analyzed via Pearson correlation. Data were transformed if needed so as to not violate normality and equal variance assumptions (see Supplementary Table S1). Supplementary data for left vs. right hemisphere differences were computed by paired *t*-test and are reported in Supplementary Table S4. Significance was set a priori at α = 0.05. In graphs, data are represented as mean ± standard error of the mean (SEM).

## 3. Results

### 3.1. Weight, Mortality, and Morbidity Following Mild TBI in Adolescent Mice

**Figure 2:**
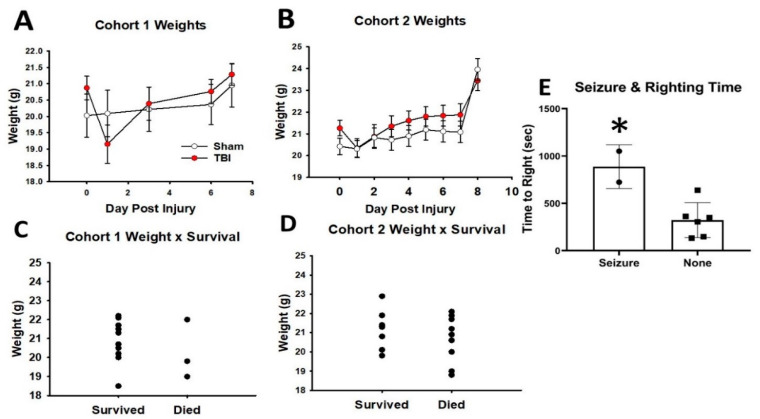
Weight, righting, and morbidity. For cohort 1 (**A**) and cohort 2 (**B**), there were no differences between sham and TBI in weight at any time up to 8 days after injury. Survival was not affected by the starting weight for cohort 1 (**C**) or cohort 2 (**D**). (**E**) The two mice who experienced seizures had significantly longer righting times (β = 0.79). * indicates *p* < 0.05.

Mice from cohort 1 were weighed preinjury, and 1, 3, 6, and 7 days post. Mice weighed between 24.2g and 18.5g preinjury, and there were no significant differences between mice in sham and TBI groups (*p* = 0.25). There was no effect of injury on weight over the days measured (*p* = 0.83). There was a main effect of the days postinjury (*p* < 0.001) and a significant interaction (*p* < 0.001). Post-hocs revealed that the significant interaction was a result of DPI 1 when sham mice weighed less than TBI, though this was not significant and likely due to the larger standard deviation (Figure 2A). Mice from cohort 2 were weighed preinjury, and each day through DPI 8. Preinjury weights ranged from 22.9g to 18.8g with no difference between the sham or TBI groups (*p* = 0.13). There was no effect of injury on weight over the days measured (*p* = 0.83). There was a main effect of DPI (*p* < 0.001) and a significant interaction (*p* = 0.02). There were no differences between groups at any DPI (Figure 2B). Preinjury weight did not impact survival in cohort 1 (*p* = 0.47) or cohort 2 (*p* = 0.31; Figure 2C,D). We did not collect enough data for cohort 1 to determine effects of righting time, but there was no correlation between weight and righting time in cohort 2 (*p* = 0.63). Mortality for cohort 1 was 33% (5/15 died). Mortality for cohort 2 was 52% (9/17 died). Finally, no mice in cohort 1 survived when a seizure was present, but two mice in cohort 2 survived, and those mice did have a significantly longer righting time than mice without a seizure (Figure 2E, *p* = 0.01). For test statistics, see Supplemental Table S3.

### 3.2. iTON Injured Mice Have a Blunted Optokinetic Response

**Figure 3:**
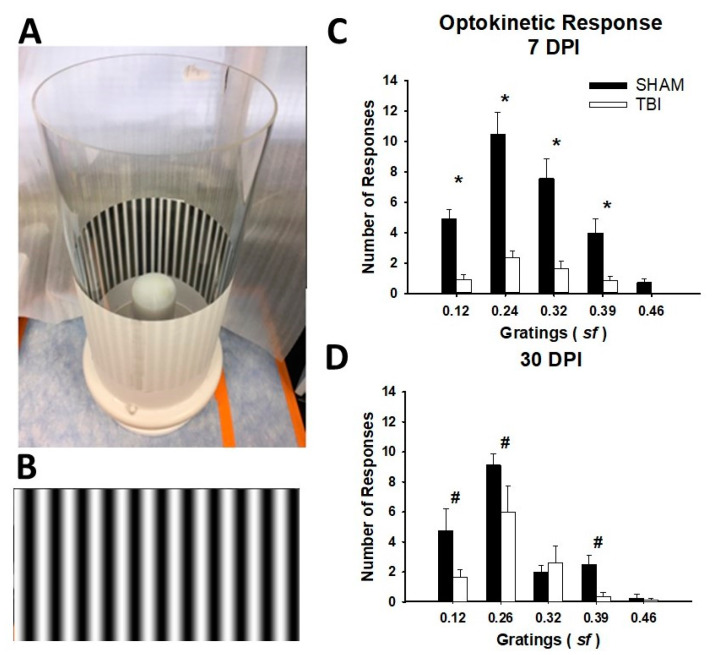
TBI/iTON mice have significantly blunted optokinetic responses. The number of responses were totaled for both left and right directions are represented as mean ± SEM for each spatial frequency analyzed. (**A**) Photograph of the behavioral testing apparatus. (**B**) Shows an example of a visual grating as used in this test. (**C**) Optokinetic responses at 7 DPI. (**D**) Optokinetic responses at 30 DPI. * *p* < 0.001 and # *p* < 0.005 vs. sham at the same grating size.

Injured mice tested from two to six days post injury displayed a significantly reduced number of optokinetic responses compared to their sham counterparts (*p* < 0.001). There was also a predicted main effect of spatial frequency (*p* < 0.001). Post-hoc analyses revealed significantly reduced responses for TON animals at all spatial frequencies (*p* < 0.001), suggesting that there was impairment in the optokinetic response and visual acuity (Figure 3C). Thirty days postinjury animals performed similarly with main effects of an injury (*p* = 0.007) and spatial frequency (*p* < 0.001). Post-hoc analyses revealed that only 0.12, 0.24, and 0.39 spatial frequencies were significantly reduced in TON-injured mice compared to sham (respectively *p* = 0.04, *p* = 0.04, and *p* = 0.004; Figure 3D). For detailed statistical results, see Supplemental Table S3.

### 3.3. Activity But Not Circadian Rhythm Is Affected in This Model of Traumatic Optic Neuropathy

Following TBI, changes to overall activity [45] and sleep disturbances [46] are commonly experienced in mice and/or humans. Traditional TBI studies often measure locomotion through assays like open field and activity monitoring to determine whether mice are less active following injury, and because we produced an injury to the visual system, we reasoned that activity monitoring could also be used to determine whether circadian rhythms were disrupted in our mice following TBI with 24/7 recording after injury. This is of particular interest to TON studies, because circadian rhythm is entrained by light cues and cells in the eye that project through the optic nerve. Thus, we also examined the brain region responsible for circadian control—the suprachiasmatic nucleus (SCN). 

**Figure 4:**
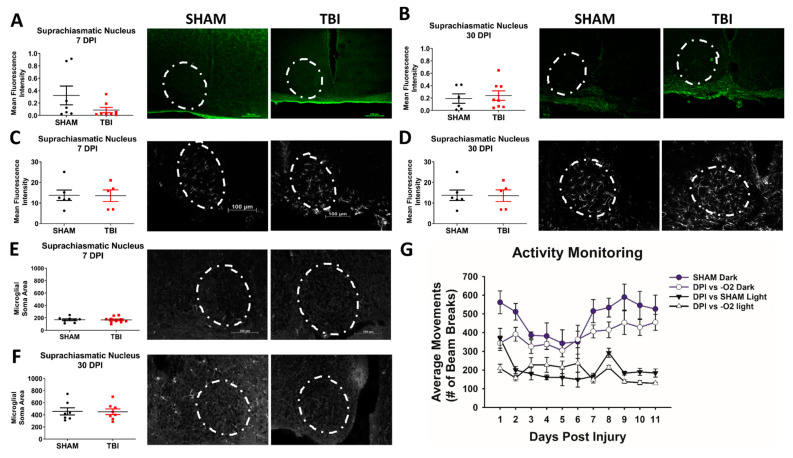
There are no circadian rhythm deficits or histological changes in the suprachiasmatic nucleus of iTON mice. Show is Fluoro-jade B (**A**,**B**), GFAP expression (**C**,**D**), and microglial soma area (**E**,**F**) in the Suprachiasmatic Nucleus (SCN). (**G**) Shows activity monitoring results. Representative photomicrographs taken at 10× magnification. White dashed lines indicate region of interest/area where measurements were taken. Scale bars indicate 100 µm and are the same across all images.

Injured mice were significantly less active than their sham counterparts (*p* < 0.001; Figure 4G), but, despite the presence of projections from the retina to the SCN, the circadian pattern of activity (i.e., more active at night) fluctuated similarly in injured and sham mice, resulting in a significant effect of light vs. dark activity (*p* < 0.001). There was no effect of day postinjury (*p* = 0.46), and reduced activity did not improve across the testing days. The lack of circadian shifts was consistent with the absence of FJ-B in the SCN (Figure 4A,B), as well as lack of significant increases in GFAP (Figure 4C,D) and no changes to microglial morphology (Figure 4E,F) at either time point. Supplementary Table S3 shows activity monitor statistics, and Supplementary Table S5 shows statistics from histological staining.

### 3.4. There Is Significant Retinal Ganglion Cell Death in TON Mice Throughout the Retina

**Figure 5:**
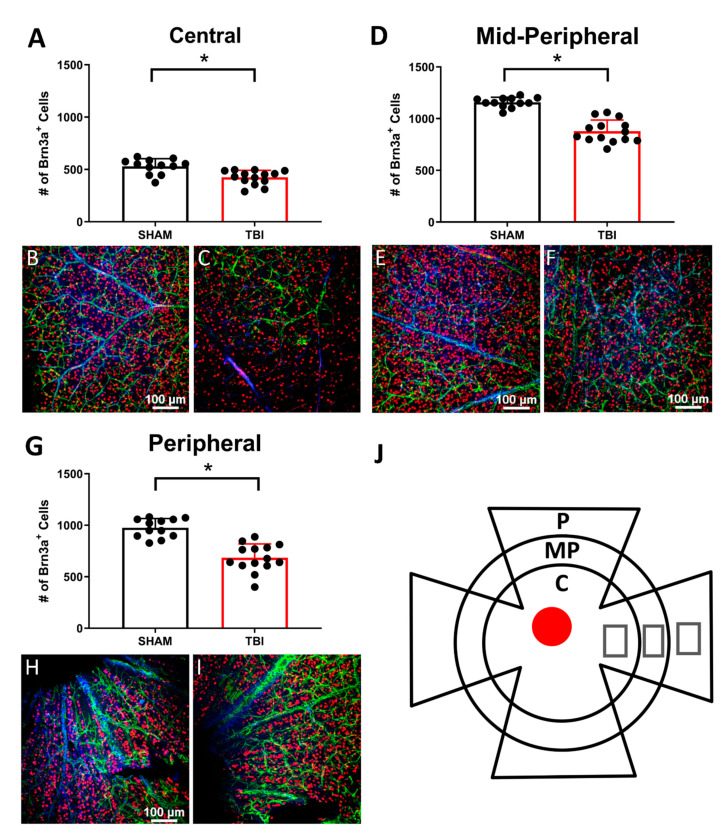
Retinal ganglion cell immunohistochemistry. Retinas of sham and TBI mice were immunolabeled with Brn3a (red), GFAP (green), and DAPI (blue). Brn3a-labeled cells were counted in 3 zones of the retina (**A**,**D**,**G**) Representative photomicrographs taken at 20× magnification of sham (**B**,**E**,**H**) and TBI/iTON mice (**C**,**F**,**I**). (**J**) Depiction of whole-mounted retina with grey squares representing photo locations. Retinas were divided into three regions: peripheral (P), mid-peripheral (MP), and central (C). The red circle indicates the location of the optic nerve. Scale bar indicates 100 μm and is the same for all images, * *p* < 0.001.

**Figure 6:**
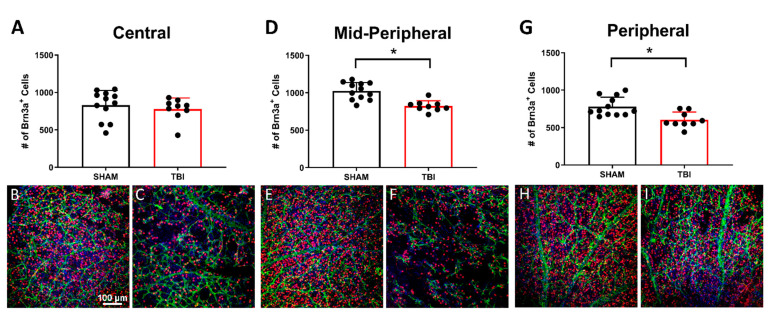
Retinal ganglion cell counts at 30 DPI. Retinas of sham and TBI mice were labeled with Brn3a (red), GFAP (green), and DAPI (blue). Cell count results are shown for center (**A**), mid-peripheral (**D**), and peripheral (**G**). Representative photomicrographs taken at 20× magnification of sham (**B**,**E**,**H**) and TBI/iTON mice (**C**,**F**,**I**). Scale bar indicates 100 μm and is the same for all images, * *p* < 0.001.

Retinal ganglion cell counts were taken 7 (Figure 5) and 30 days postinjury (Figure 6) to determine whether this injury was localized to the optic nerve. The marker Brn3a was chosen, as it has been shown to label the majority of RGCs with comparable success to FluoroGold tracing methods in other models of retinal axonal degeneration [47,48], and Brn3a-positive cells also predominantly project to regions where we have been shown worse histological outcomes (i.e., thalamic and collicular structures) [49]. To acquire an accurate sampling of RGC densities throughout the retina, counts were taken in three zones, as explained in our Methods section (Figure 5J). At 7 DPI, there were significantly fewer RGCs in TON injured (*n* = 14) mouse retinas compared to shams (*n* = 12) in the peripheral (*p* < 0.001), mid-peripheral (*p* < 0.001), and central regions (*p* < 0.001). Cell counts were also significantly reduced in TON (*n* = 12) compared to sham (*n* = 9) mice 30 DPI only in the peripheral (p 0.002) and mid-peripheral (*p* < 0.001) quadrants but not in the center (*p* = 0.4). Injured mice also had significantly elevated Caspase3 7 DPI (*p* < 0.001; Figure 10b), suggesting active cell death occurs up to at least 7 DPI. This elevation was no longer significant at 30 DPI (*p* = 0.7; Figure 11b). Of further note, we stained for astroglial activation (with GFAP), but the staining was inconsistent even in the control animals likely due to the nature of whole-mount staining, so we could not quantify it. Supplementary Table S5 includes statistical data for Brn3a staining and Supplementary Table S6 for caspase-3.

### 3.5. Injured Mice Have Significantly Increased Axonal Degeneration Throughout the Primary Visual System

**Figure 7:**
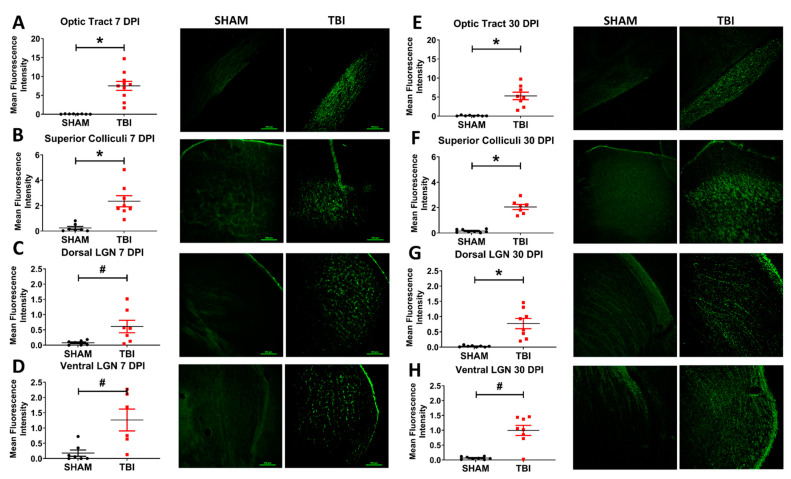
Neurodegeneration in the optic system 7 (**A**–**D**) and 30 (**E**–**H**) DPI. Staining appears punctate rather than somatic, consistent with primarily axonal staining. Representative photomicrographs taken at 10× magnification with a scale bar indicating 100 μm; this applies to all images. * *p* < 0.001 and # *p* <0.05.

FJ-B staining for degeneration showed a pattern of punctate staining indicative of axonal degeneration throughout the majority of retinal ganglion cell projection targets [23,44] (Figure 7). Areas examined were the optic tract (OT), superior colliculi (SC), ventral lateral geniculate nucleus (vLGN), dorsal lateral geniculate nucleus (dLGN), and suprachiasmatic nucleus (SCN). No FJ-B staining was present in the visual cortex of either sham or TON mice. There were no differences in histological staining between left and right hemispheres (Supplementary Table S4), indicating a uniform and bilateral injury to the optic system. At 7 DPI, injured mice presented with increased degeneration (as calculated by the mean fluorescence intensity) in the optic tract (TON *n* = 8, sham *n* = 10; *p* < 0.001), vLGN (TON *n* = 7, sham *n* = 7; *p* = 0.005), dLGN (TON *n* = 7, sham *n* = 7; *p* = 0.02), and SC (TON *n* = 7, sham *n* = 8; *p* < 0.001). There was no staining in the suprachiasmatic nucleus (TON *n* = 8, sham *n* = 7; *p* = 0.1; Figure 4A). This degeneration was also present 30 DPI in OT (TON *n* = 8, sham *n* = 8; *p* < 0.001), vLGN (TON *n* = 8, sham *n* = 8; *p* = 0.005), dLGN (TON *n* = 8, sham *n* = 8; *p* < 0.001), and SC (TON *n* = 7, sham *n* = 8; *p* < 0.001) but not SCN (TON *n* = 8, sham *n* = 6; *p* = 0.5; Figure 4B). Interestingly vLGN and dLGN were also significantly different within TON mice (*p* = 0.03), with the vLGN of injured mice having a higher mean fluorescence intensity (*M* = 0.99, *SD* = 0.17) than dLGN 7 DPI (*M* = 0.77, *SD* = 0.17). Supplementary Table S5 displays statistical test results.

### 3.6. There Is Microglial Activation in Some Downstream Optic Subcortical Thalamic Targets

**Figure 8:**
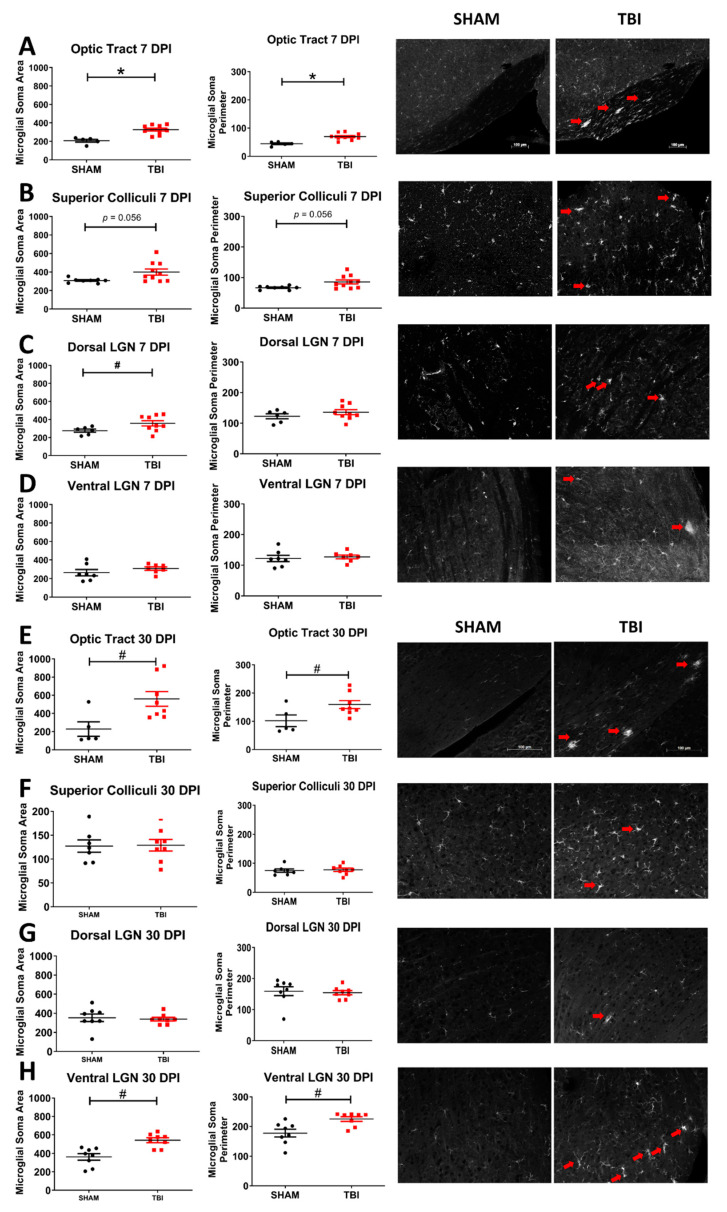
Morphologic evidence of microglial activation in the optic system is present 7 (**A**–**D**) and 30 (**E**–**H**) DPI. These morphologic changes are particularly predominant in the OT but can also clearly be seen in other regions (red arrows). Representative photomicrographs taken at 10× magnification, scale bar 100 μm. # *p* < 0.05. Higher magnification images can be found in Supplementary Figure S3, * *p* < 0.001.

The same thalamic and brainstem targets of retinal ganglion cells were analyzed for microglial activation by Iba-1 fluorescent immunostaining 7- and 30- days post injury (Figure 8). Microglial soma area and perimeter were utilized to assess morphological changes in the microglia that are known to become enlarged and ameboid in response to CNS damage, such as in TBI; such morphologic changes are considered a marker of neuroinflammation [50,51]. At 7 DPI, there was a significantly increased soma area (TON *n* = 5, sham *n* = 10; *p* < 0.001) and perimeter (*p* < 0.001) in the optic tract, and the area (TON *n* = 9, sham *n* = 6; *p* = 0.03) but not perimeter (*p* = 0.29) was significantly increased in dLGN. There was not a significant change in microglial morphology in the superior colliculi (area *p* = 0.06, perimeter *p* = 0.06), vLGN (area *p* = 0.3, perimeter *p* = 0.7), or suprachiasmatic nuclei (area *p* = 0.9, perimeter *p* = 1; see Figure 4E). 

At 30 DPI, optic tract microglia are also significantly different from sham in the area (TON *n* = 8, sham *n* = 5; *p* = 0.002) and perimeter (*p* = 0.04). Ventral lateral geniculate nuclei area (TON *n* = 8, sham *n* = 8; *p* = 0.002) and perimeter (*p* = 0.01) are significantly higher in injured mice but not in the dorsal LGN (area *p* = 0.8, perimeter *p* = 0.5). There was also not a significant change in the microglial morphology in the superior colliculi (area *p* = 0.9 and perimeter *p* = 0.8) or SCN (area: *p* = 0.9; perimeter: *p* = 0.8; Figure 6F). Supplementary Table S5 displays the statistical test results.

### 3.7. There Is Astrogliosis after TBI Throughout the Visual System Indicative of Traumatic Optic Neuropathy 

**Figure 9:**
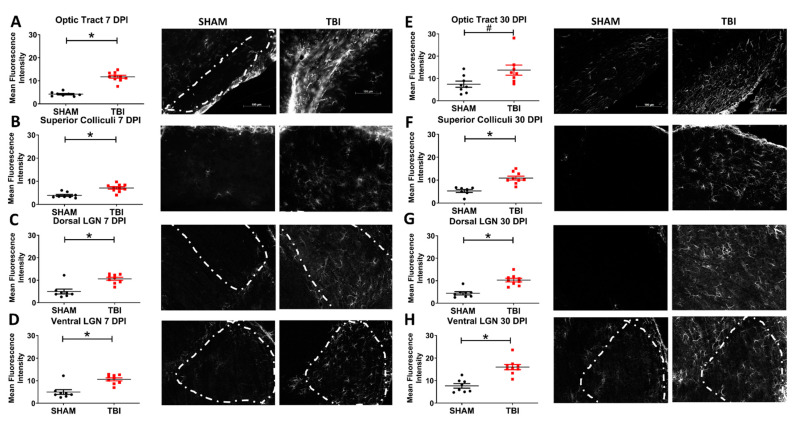
Astrogliosis of the optic system in iTON mice 7 (**A**–**D**) and 30 (**E**–**H**) DPI. White dashed lines mark the areas analyzed in sham and iTON mice where it is more difficult to visualize the location of the nuclei. Representative photomicrographs taken at 10× magnification, scale bar 100 μm. * *p* ≤ 0.001 and # *p* < 0.05.

Optic tract astrogliosis was also seen, with significantly elevated expression of GFAP 7 days postinjury (TON *n* = 8, sham *n* = 8; *p* < 0.001; Figure 9). Moreover, there is reactive gliosis present in the vLGN (TON *n* = 9, sham *n* = 9; *p* < 0.001), dLGN (TON *n* = 8, sham *n* = 9; *p* = 0.001), and superior colliculi (TON *n* = 8, sham *n* = 10; *p* < 0.001) of injured mice 7 DPI but not in the suprachiasmatic nuclei (TON *n* = 5, sham *n* = 6; *p* = 0.9; Figure 6C). These effects are also seen 30 DPI with significantly increased gliosis in the optic tract (TON *n* = 8, sham *n* = 8; *p* = 0.02), vLGN (TON *n* = 8, sham *n* = 9; *p* < 0.001), dLGN (TON *n* = 8, sham *n* = 9; *p* < 0.001), and SC (TON *n* = 7, sham *n* = 9; *p* < 0.001) of TBI mice but, again, not the SCN (*p* = 0.44; Figure 6D). Supplementary Table S5 displays the statistical test results.

### 3.8. ER Stress Markers Are Elevated after Experimental Head Trauma 

**Figure 10:**
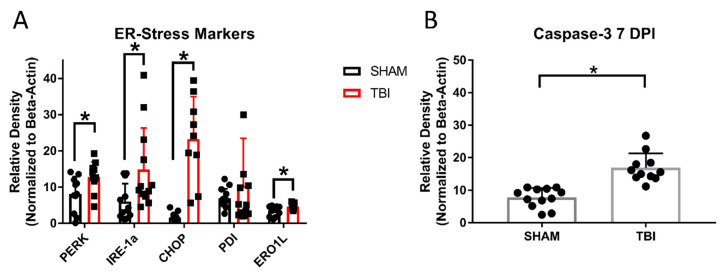
Markers of apoptosis and endoplasmic reticulum stress in the retinas of TBI/iTON mice 7 DPI. (**A**) Shows markes implicated in ER stress, (**B**) shows elevated Caspase-3 (an apoptotic marker). Protein measurements were normalized to beta-actin. * *p* < 0.05.

**Figure 11:**
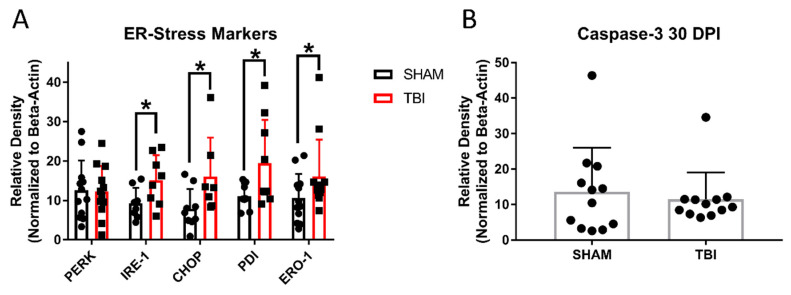
Markers of endoplasmic reticulum stress (**A**) and caspase-3 expression (**B**) at 30 DPI. * *p* < 0.05.

There are three major ER stress pathways in mammals. Two of these pathways function around the IRE-1α and PERK receptors with downstream effectors, including PDI, CHOP, and eRO1L. We performed Western blotting on the retinal protein extracted 7 and 30 days postinjury. At 7 DPI (Figure 10A), there were significantly elevated levels of IRE-1α (TON *n* = 14, sham *n* = 14; *p* = 0.02), PERK (TON *n* = 14, sham *n* = 11; *p* = 0.006), ERO1-L (TON *n* = 11, sham *n* = 12; *p* = 0.02), and CHOP (TON *n* = 17, sham *n* = 9; *p* < 0.001) but not PDI (*p* = 0.4). At 30 DPI (Figure 11A), the ER stress markers remained elevated in TON mice for IRE-1α (TON *n* = 8, sham *n* = 8; *p* = 0.04), ERO1-L (TON *n* = 12, sham *n* = 12; *p* = 0.04), and CHOP (TON *n* = 8, sham *n* = 8; *p* = 0.03). Interestingly, PERK activation was no longer significantly elevated (*p* = 0.9) but PDI was (TON *n* = 9, sham *n* = 8; *p* = 0.04). See Supplementary Table S6 for statistical analysis reports and Supplementary Figures S4 and S5 for representative raw blot images.

## 4. Discussion

Utilizing a mild weight-drop injury we produced a novel model for analyzing indirect traumatic optic neuropathy within the context of mild-to-moderate traumatic brain injury. This model recapitulates key features of TON without direct penetration of the optic nerve, is easily replicated, and shows evidence of injury from the molecular to the behavioral level for at least 30 days after an injury. While we have previously shown similar results to those described here in adult mice [23], the current study examines more features of TON and extends our adult findings to adolescent mice, thus further validating this as a replicable model of TON across ages and multiple study groups. We have shown that indirect TON results in injury not only to the optic nerve, with associated retinal ganglion cell death, but also shows effects in more downstream subcortical and brainstem targets of RGC axons, including the dorsal and ventral lateral geniculate nuclei and superior colliculi but not the suprachiasmatic nucleus or visual cortex. Lack of differences in circadian activity between sham and injured mice over the first 11 days after injury is consistent with absence of histologic damage in the suprachiasmatic nuclei through 30 DPI. We additionally provided evidence that this injury affects the optokinetic response and that degeneration, astrogliosis, and microglial activation are accompanied by elevated endoplasmic reticulum stress markers up to 30 days postinjury. 

We first attempted to extend our previous findings by adding a behavioral measure of impaired visual function. We, therefore, exploited a common behavioral assay used in models of optic degeneration, retinitis pigmentosa, and other optic neuropathies for the optokinetic response, which correlates with visual acuity [38]. TON mice both 7 and 30 DPI experience a significantly blunted OKR compared to their uninjured counterparts. The optokinetic response is a critical visual function, as it allows one to perceive and follow objects as they cross the field of vision, as well as the proper function of smooth eye movements and other reflex eye movements [52]. OKR deficits could also indicate a decline in visual acuity, as one needs to be able to differentiate a moving object to follow it [38]. In conjunction with previously reported optical coherence tomography and flash electroretinogram data, reduced OKR is likely due to thinning of the retinal layers, reduced RGC functioning and/or death, and persistent degeneration in visual centers of the brain [24]. In the future, it would be interesting to directly correlate this behavioral outcome with pattern electroretinogram.

In addition to vision deficits, we also showed a significant reduction in the number of retinal ganglion cells both 7 and 30 days after injury. At 7 DPI, there were significantly reduced RGCs across all three retinal regions analyzed, but at 30 DPI, this was only true in the peripheral and mid-peripheral regions of iTON mice. Our analysis of the caspase-3 apoptotic marker suggests the presence of apoptotic cells early after injury but not at 30 DPI. Thus, RGC death is an early consequence of indirect traumatic optic neuropathy that results in retinal cell apoptosis. The continued presence of cells expressing apoptotic markers at 7 DPI may suggest a window of several days after injury for possible therapeutic interventions targeting this process. 

This shift away from apoptosis may also be indicative of changing responses in the unfolded protein response. There is a significant body of literature on the effects of ER stress in many models of optic neuropathies [13], but relatively little is known about the role of ER stress in the pathology of traumatic optic neuropathy. For instance, the downstream apoptotic effector of the ER stress pathway, CHOP, was upregulated in TON mice at 7 DPI. This suggests that, after injury, cellular stress is high, and the unfolded protein response appears to lead to apoptosis rather than refolding, even in the acute phase after TBI [53]. In contrast, PDI is not elevated 7 days postinjury but is at 30 DPI. PDI has two functions in the ER stress pathway as a redox-sensing activator of IRE-1α and PERK receptors and as a downstream nuclear chaperone of the UPR-PERK pathway that promotes the refolding of misfolded proteins [53,54,55]. This lack of PDI upregulation relatively early after injury may indicate that it is not acting as an ER stress agonist in this injury model and/or that the level of stress is too high for the cell to push for repair over apoptosis, which is supported by our CHOP and caspase-3 data 7 DPI. It could also be that an acute period of activation occurred prior to our earliest measurements at 7 DPI. Moreover, the delayed increase in PDI may favor a role as a chaperone for protein repair, which could indicate the potential for therapeutic strategies within a month postinjury, where RGCs were no longer dying but recovering from the injury. 

We also analyzed the retinas of sham and injured mice to determine to what degree major ER stress pathways were activated by the experimental traumatic optic neuropathy. Indeed, two of the three major pathways (IRE-1α and PERK) were upregulated at 7 DPI, but only the IRE-1α pathway remained elevated at 30 DPI. Additionally, the elevation of ERO-1L further supports the conclusion of elevated ER stress and, also, implicates oxidative stress, since this protein is active in both the oxidative stress and ER stress pathways [56]. Veritably, the recent literature suggests that the oxidative and ER stress pathways are interconnected, although the mechanisms are not fully clear, at least within TBI studies [13,57,58,59]. Additionally, we are the first to propose this connection between reactive oxygen species, which were present within 30-90 min after a sonic blast [24], and ER stress in an optic neuropathy model and are the first to show that redox stress is likely to persistent far longer than 90 min. We acknowledge that the use of beta-actin expression as a “house-keeping” gene to normalize semi-quantitative Western blot results has become more controversial, due to the possibility of changes in actin expression. However, there was no difference in actin expression between TBI and sham groups in either the 7-day or 30-day cohort. Nevertheless, future studies still need to find a way to separate these stress responses and determine whether these proteins are working through united or separate mechanisms. Additionally, other mechanisms of cell death like Muller (microglial) cell and astroglial activation in the retina also need to be explored.

Our data are also consistent with RGC loss due to degeneration, which is initiated by injury to the intracanalicular portion of the optic nerve [23]. Positive FJ-B staining in the optic tract was present at 7 and 30 days postinjury. Of note, the pattern of histologic staining for degeneration seen in these experiments (Figure 7) was punctate and morphologically consistent with axons. This suggests that there were degenerating axons in the regions noted. Supporting evidence for similar axonal injury in the optic nerve has also been found in blast models [22,25,26] and midline fluid percussion injury, as well as targeted DIA to the optic nerve [60]. We did not see evidence of trans-synaptic neuron loss, as there was no evidence of cell soma degeneration in brain areas with trans-synaptic optic nerve projections (e.g., visual cortex). We also showed that markers of neuroinflammation and astrogliosis were elevated 7 and 30 DPI in the optic nerve/tract, suggesting that the pathologic processes were yet resolved at these times. These results suggest that degenerative processes may persist for long periods after injury and are supported by indirect and direct models of optic nerve injury [24,61], although long-term functional consequences are not yet fully elucidated. It has also been shown that gliosis surrounding axonal damage likely progresses along the axons to its far-reaching projections [62]. Thus, we examined multiple projection targets of the optic nerve for evidence of degeneration.

Indeed, there is increased degeneration and gliosis, but not microglial activation, in the superior colliculi at 7 and 30 DPI. These data indicate that the axonal degeneration seen in the anterior optic tract extends to the midbrain colliculi. Further, because the superior colliculus plays a role in the integration of visual attention, saccadic eye movement, and localization of attention shifts to new stimuli; the deficits in optokinetic performance may be explained in part by these histological findings. Future studies should more closely examine other areas involved in the OKR, like those of the accessory optic system, as well as attempt to test these distinct visual functions. The fact that there is no microglia reactivity at 7 or 30 DPI suggests that the pathophysiology of injury here may be driven more by reactive gliosis rather than neuroinflammation. Recent studies probing the timing of astrocytic vs. microglial activation following TBI have suggested that microglia are present closer to the site of injury initially while downstream locations favor astrocyte activation, with microglial inflammation occurring even years after the initial insult [63]. Thus, it is possible that the difference in microglial morphologic changes between the lateral geniculate nuclei and superior colliculi could be due to the fact that the SC is physically further from the site of injury. 

We visualized neurodegeneration, gliosis, and activated microglia in thalamic projections of the optic tract in the LGN. The LGN is anatomically divided into ventral and dorsal subnuclei. This division is important, because the vLGN is more directly connected to the superior colliculi and has also been implicated in the optokinetic response and circadian rhythm, while the dLGN is known to project primarily to the visual cortex in both rats and cats [64,65]. At 30 DPI, both vLGN and dLGN also have significantly greater degeneration than found in sham animals, and the vLGN has significantly higher FJ-B intensity than the dorsal geniculate nuclei in TON mice. Thus, the lack of degeneration in the visual cortex despite positive staining in the dLGN may indicate that there is no significant trans-synaptic degeneration at the time points analyzed. Additionally, there is a significantly increased soma area of the microglia in the dLGN but not the ventral LGN 7 DPI. 30-DPI; however, there is significantly changed microglial morphology in the vLGN but not the dLGN. While we are uncertain as to why the two regions differ at different time points, it does seem that projections to the dorsal LGN and ventral LGN respond to this injury differently. One possibility may be differences in the susceptibility of retinal cell subtypes to injury, as this has been shown in other optic nerve models [66,67]. Moreover, astrogliosis is more consistent across both lateral geniculate nuclei divisions, which may suggest that astroglia are playing a larger role in the detrimental effects of the injury sub acutely.

Prolonged elevation of astrocyte activity, as well as persistent microglial activation, can result in protective and/or pernicious outcomes. It has recently been disputed whether gliosis serves more immediate protective functions through the formation of a gliotic scar at the site of injury or through the release of growth factors (TGFβ) and cytokines (IL-6), which support the repair of injured tissue and reduce neuroinflammation, or even by engulfing the injured tissue and inhibiting contact-induced apoptosis [68,69,70] or if the consequences of activation are more deleterious. For example, the gliotic scar prevents axonal regrowth and repair [68], but the removal of proliferating glia (i.e., glia that form the gliotic scar) results in increased neurodegeneration to the impact site, so astrocytes’ roles are not yet fully understood [71]. Still, other research suggests that it is the milieu of microglia and astrocytes together that is critical for proper improvement after injury. Microglia are signaled in response to gliosis but can also respond to injury in more acute periods, preceding development of the gliotic scar [63]. Fitch and Silver further argue that proximity to the injury may determine the dichotomous roles of astrocytes, such that those inhibiting axon regrowth may be closer to the injury and those releasing beneficial growth factors may be more distant [62]. Identifying activated astrocytes by the GFAP content, as was done in this study, would not differentiate these activities, so future studies are needed to differentiate between these possibilities. 

Though the vLGN is implicated in circadian rhythm regulation [72] there were no indications of circadian dysfunction in this injury model. There was also no evidence of degeneration, inflammation, or gliosis in the suprachiasmatic nuclei, nor were there any shifts in the activity patterns measured over 11 DPI, despite animal TBI studies showing shifts in both the motor activity and orexin levels 3 days postinjury [46], as well as the acute loss of cortisol-mediated circadian rhythm in adults 18–65 [73] and increased melatonin-mediated shifts in children after severe TBI [74]. It is also possible, though, that circadian shifts may take longer to develop, as has been true in human populations ranging from 4 months to 2 years after injury [74]. This may indicate that a subset of RGCs, known as intrinsically photosensitive RGCs (or melanopsin-containing RGCs), that project directly to the SCN and help mediate light entrainment of circadian rhythms [75], may be less susceptible to injury in this model. Indeed, this is the case in the optic nerve crush and transection models [66]. It is also possible that this difference in apparent injury susceptibility between RGC populations is due to the part of the cross-sectional area of the optic nerve in which these fibers run, since, in at least some species, there is a consistent, likely retinotopic, organization of optic nerve fibers within the nerve [76]. In this case, some fibers in the optic nerve could possibly be protected by their relative position in the nerve (for example, by being in the center of the nerve and, thus, partially shielded from impact). However, in our studies, degenerating axons appear to be distributed throughout the optic tract diameter (Figure 7A,E) rather than having a discernible geometric pattern within the tract. Future studies will need to analyze these photosensitive RGCs, as well as photoreceptors, to determine whether this might explain the lack of damage to the SCN.

## 5. Conclusions

In summary, we showed that this closed head trauma model in adolescent mice leads to reproducible optic tract injury, and this can be used as a model of indirect traumatic optic neuropathy. We described significant changes to the optokinetic response that are present early and late after injury. There is clear retinal ganglion cell loss 7 and 30 days postinjury and active apoptosis at 7 DPI. There is axonal degeneration, neuroinflammation, and gliosis in the optic tract, superior colliculi, and Lateral Geniculate Nuclei. The elevation of multiple early and late ER stress markers suggests that ER stress is a possible pathologic mechanism for the ongoing degeneration. There is also evidence of both ER stress and oxidative stress through persistent ERO-1L elevation. This model shows promise for ongoing study into the pathologic mechanisms and potential treatment strategies for indirect TON associated with head trauma.

## Data Availability

The data supporting the reported results are found in the figures, tables, and supplementary figures/tables.

## Supplementary Materials

The following Supplementary Materials can be found at https://www.mdpi.com/article/10.3390/cells10050996/s1: Figure S1: Snapshot of Optomotor Machine Recording, Table S1: Data transformations used to pass normality, Figure S2: Secondary antibody-only control for brn3a immunohistochemistry, Table S2: Weight and morbidity statistical results, Table S3: Behavioral statistical results, Table S4: Comparison of left vs. right hemisphere structures, Table S5: Immunofluorescence statistical results, Figure S3: Larger images of IBA-1-labeled cells, Table S6: Western blotting statistical results, Figure S4: Representative raw Western blot films at 7 DPI, and Figure S5: Representative raw Western blot films 30 DPI.
