# Supplementary material for: Traumatic Optic Neuropathy Is Associated with Visual Impairment, Neurodegeneration, and Endoplasmic Reticulum Stress in Adolescent Mice
Source: Cells. 2021 Apr 23;10(5):996. doi: 10.3390/cells10050996 (PMC8146890; doi:10.3390/cells10050996)
Supplement: Supplementary file 1 [file cells-10-00996-s001.zip › cells-1138752-supplementary.pdf]

Supplementary Materials

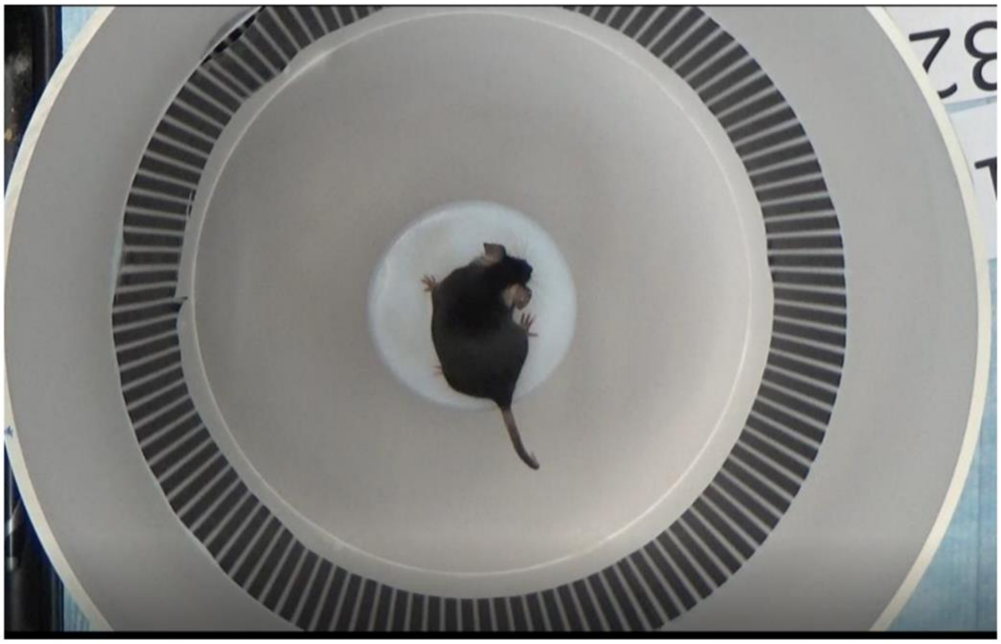

Supplementary Figure S1. Snapshot of OMM Recording. This is a screenshot of one of our behavioral recordings. Mice were clearly visible against the white background. The camera was placed roughly 2.5 feet above the machine and zoom was used to visualize the entire field inside the drum while also producing a close image for easier scoring.

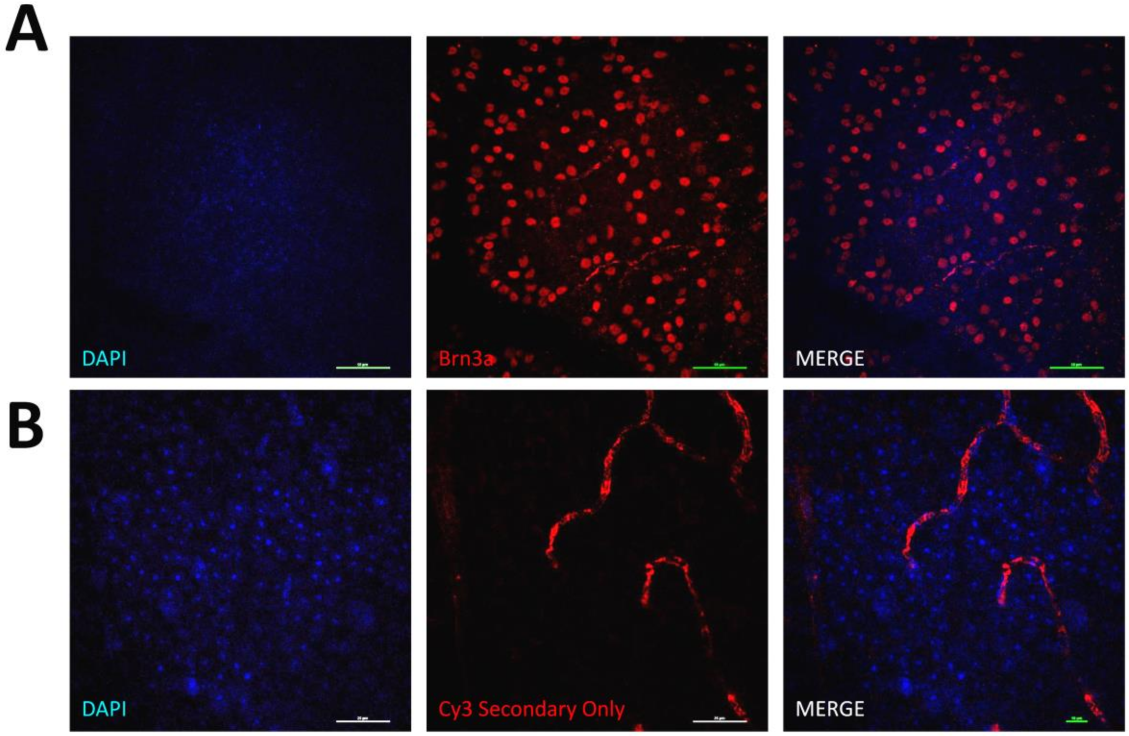

Supplementary Figure S2. Secondary-only control for Brn3a antibody. Because our Brn3a antibody was a mouse monoclonal antibody, we included a secondary only control for which all processes of immunostaining remained the same save for incubation with primary Brn3a which was replaced with only blocking solution. (A) Shows a retina that underwent normal IHC with Brn3a – from left to right DAPI only, Brn3a only, merged image. (B) Depicts the control retina for which we see no Cy3-positive labeling overlapping with or around the DAPI nuclear label. Images were taken at 40x magnification and scale bars represent 100µm.

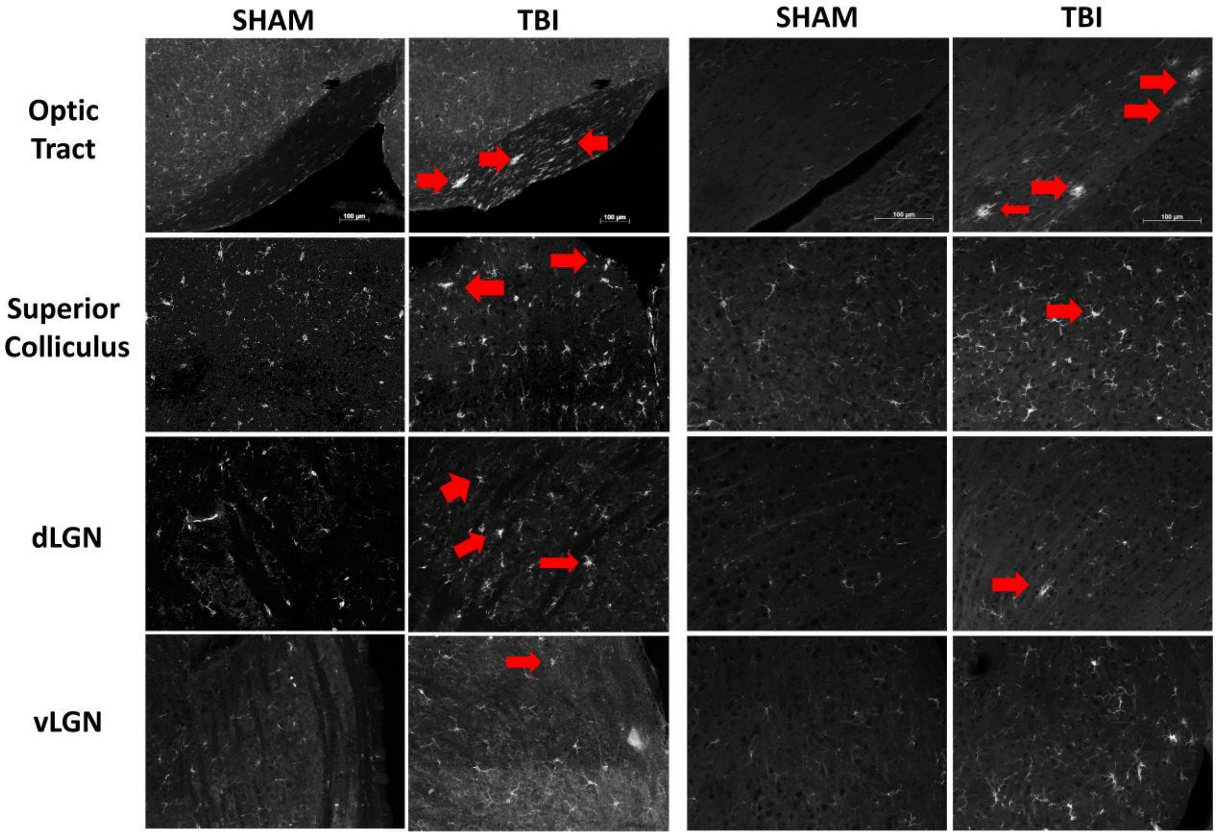

Supplementary Figure S3 Larger images of IBA-1 labeled cells in Optic Tract, Superior Colliculus, and Lateral Geniculate Nuclei. Larger soma size are indicative of more activated microglia and, therefore, possible neuroinflammation. This morphology is particularly evident in the optic tract. Red arrows identify larger microglia compared to sham animals. Scale bars in OT images correspond to all other images and represents 100 µm.

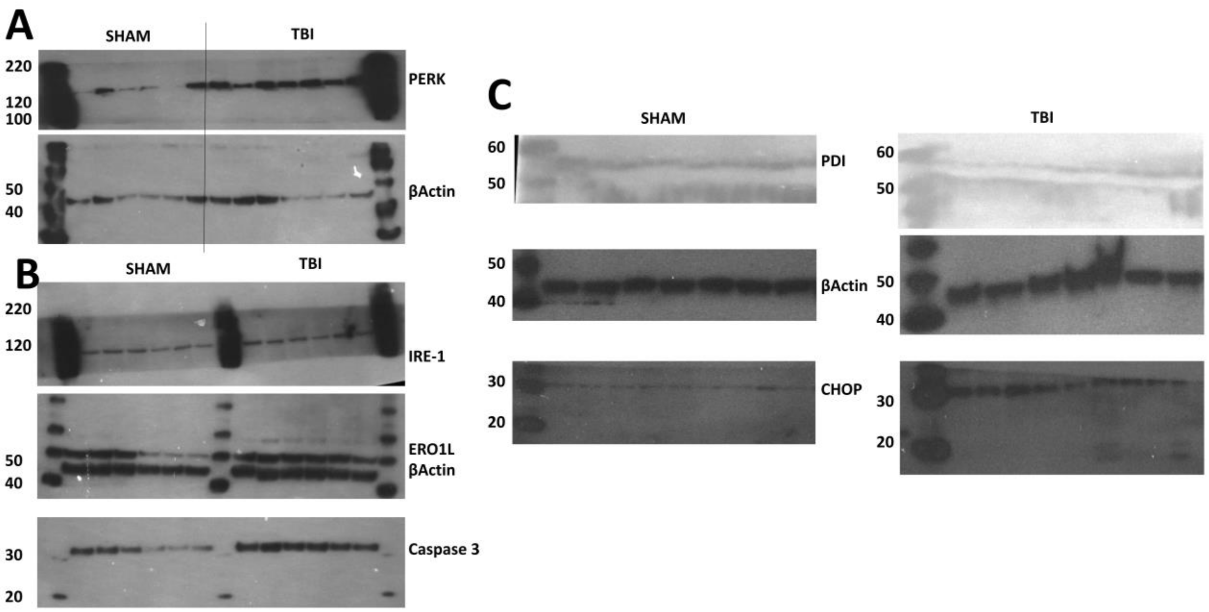

Supplementary Figure S4. Representative Raw Western Blot Films 7 DPI. Reviewers were given whole, unedited images before publication. (A) PERK and βActin were stained separately, but both were from the same membrane that was cut horizontally. (B) IRE-1 and Cas3 were stained individually while ERO-1 and actin were stained together. All were from the same membrane that was cut horizontally. (C) Sham and TBI were separated but one animal (well 1 after ladder) remained the same on both blots to normalize across membranes and then each animal was normalized to its respective actin. CHOP and βActin were stained separately, then the actin membrane was stripped and stained for PDI. Images represented here were cropped to emphasize the bands reported and to show differences between groups analyzed.

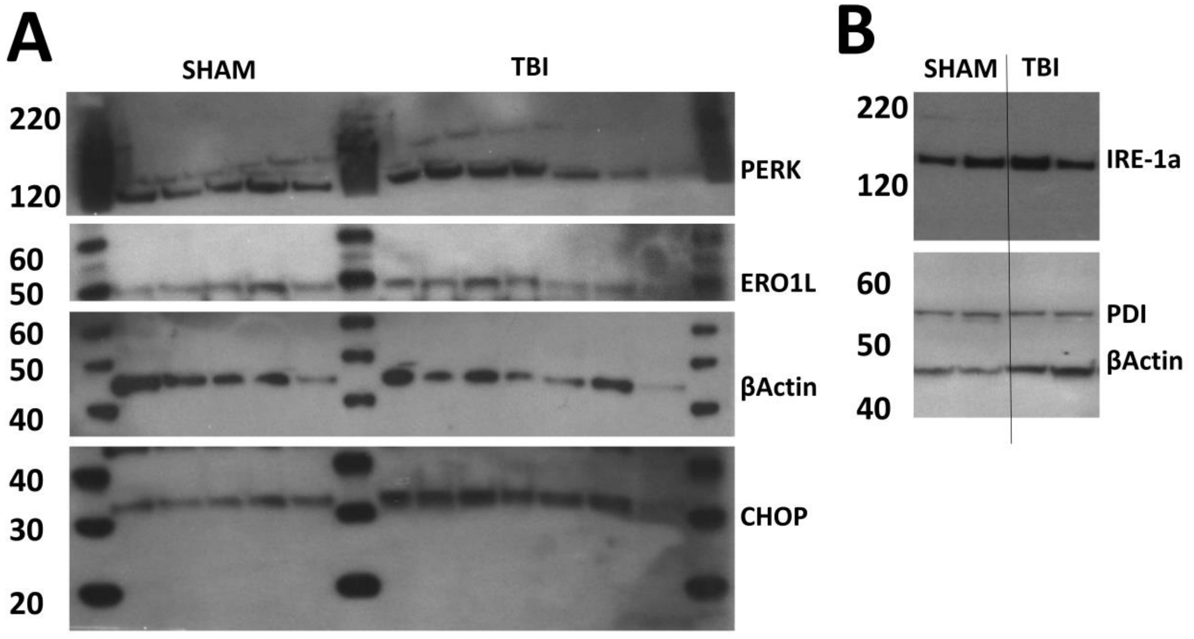

Supplementary Figure S5. Representative Raw Western Blot Films 30 DPI. Reviewers were given whole, unedited images before publication. (A) PERK, ERO-1L, and CHOP were stained from the same membrane cut horizontally. The membrane was stripped and βActin was stained for. (B) IRE-1, PDI, and βActin were stained from the same membrane cut horizontally. Images represented here were cropped to emphasize the bands reported and to show differences between groups analyzed.

Supplementary Table S1. Data transformations used to pass normality.						
**Stain**	**FJ**					
**Region**	**OT**	**dLGN**	**vLGN**	**SC**	**SCN**	
**7 DPI**	sqrt	-	-	sqrt	log	
**30 DPI**	log	-	-	-	log	
**Stain**	**Iba-1**					
**Region**	**OT**	**dLGN**	**vLGN**	**SC**	**SCN**	
**7 DPI**	-	-	-	reciprocal	-	
**30 DPI**	log	-	-	-	-	
**Stain**	**GFAP**					
**Region**	**OT**	**dLGN**	**vLGN**	**SC**	**SCN**	
**7 DPI**	-	-	-	-	-	
**30 DPI**	reciprocal	-	-	-	-	
**ER Stress**						
**Region**	**IRE-1**	**PERK**	**ERO1L**	**PDI**	**CHOP**	**Caspase**
**7 DPI**	log	log		log		
**30 DPI**			log	sqrt	log	log

Supplementary Table S1. If an analysis did not pass normality, we proceeded with the following steps: (1) determine skew of data, (2) if data was skewed positively, log10 then sqrt transformations were applied, (3) if data were negatively skewed, square then log10 transformations were applied, (4) if these three failed, reciprocal transformation was applied, (f) if all failed, a non-parametric test was used. A blank square indicates no transformation was used. Sqrt = square root, log = log(10).

Supplementary Table S2. Weight and Morbidity Statistical Results					
**Cohort**		**Analysis**		**Test Statistic**	**p-value**
**Cohort 1**	**Weight**	**2way RM ANOVA**	**Injury**	$F_{1,89}$=0.05,	0.25
			**Day**	$F_{4,89}$=5.15,	<0.001
			**Interaction**	$F_{4,89}$=7.23,	<0.001
**Cohort 2**	**Weight**	**2way RM ANOVA**	**Injury**		0.83
			**Day**	$F_{4,89}$=5.15,	<0.001
			**Interaction**	$F_{4,89}$=7.23,	0.02
**Cohort 2**	**Seizure & Righting Time**	**t-test**		t(6)=-3.59	0.01

Supplementary Table S2. There were no effects of injury on weight up to 8 DPI in either cohort. Only one variable produced a significant difference when examining morbidity – of the two mice in cohort 2 that survived TON with a seizure, they both took significantly longer to right (M=887.5sec, SD=231) than those without a seizure (M=322.5 sec; SD= 184.1).

**Supplementary Table S3. Behavioral Statistical Results**					
**Cohort**		**Analysis**		**Test Statistic**	**p-value**
**Cohort 1**	**Optokinetic Response**	**2way RM ANOVA**	**Injury**	$F_{1,\ 123} = 45.5$	<0.001
			**Spatial Frequency**	$F_{4,123} = 32.8$	<0.001
			**Interaction**	$F_{4,124} = 3.25$	0.01
**Cohort 2**	**Optokinetic Response**	**2way RM ANOVA**	**Injury**	$F_{1,79} = 9.9$	0.007
			**Spatial Frequency**	$F_{4,79} = 22.6$	<0.001
			**Interaction**	$F_{4,79} = 1.73$	0.2
**Cohort 2**	**Activity Monitor**	**3way RM ANOVA**	**Injury**	$F_{1,43} = 4.7$,	0.04
			**Light**	$F_{1,43} = 147.5$,	<0.001
			**Day**	$F_{1,43}= 1.0$,	0.46
			**Interaction**	none	none

Supplementary Table S3. ANOVA test statistics for optomotor and activity monitoring behavior assays. Data pairs with figures 3 and 6.

**Supplementary Table S4. Immunofluorescence and Immunohistochemical Statistical Results**				
**Cohort**	**Stain**	**Region**	**t (or U)**	**p-value**
**Cohort 1**	**Brn3a (RGCs)**	**Peripheral**	6.3	<0.001
		**Mid-peripheral**	8.2	<0.001
		**Central**	-4.0	<0.001
	**FJ**	**Optic Tract**	-9.4	<0.001
		**Dorsal LGN**	-2.6	0.02
		**Ventral LGN**	-3.1	0.005
		**Superior Colliculi**	-6.0	<0.001
		**Suprachiasmatic Nucleus**	1.61	0.13
	**IBA-1 Area**	**OT**	-4.5	<0.001
		**dLGN**	-2.41	0.03
		**vLGN**	-1.12	0.3
		**SC**	U=18	0.06
		**SCN**	0.11	0.91
	**IBA-1 Perimeter**	**OT**	-4.2	<0.001
		**dLGN**	-1.01	0.29
		**vLGN**	-0.42	0.7
		**SC**	U=18	0.06
		**SCN**	-0.00	1.0
	**GFAP**	**OT**	-9.8	<0.001
		**dLGN**	-3.9	0.001

		**vLGN**	-5.5	<0.001
		**SC**	-4.6	<0.001
		**SCN**	0.05	0.9
**Cohort 2**	**Brn3a (RGCs)**	**Peripheral**	3.5	0.002
		**Mid-peripheral**	4.7	<0.001
		**Central**	0.9	0.4
	**FJ**	**OT**	-9.1	<0.001
		**dLGN**	-4.4	<0.001
		**vLGN**	U=6	0.005
		**dLGN vs vLGN**	2.4	0.03
		**SC**	-9.7	<0.001
		**SCN**	-0.76	0.5
	**IBA-1 Area**	**OT**	4.1	0.002
		**dLGN**	0.32	0.76
		**vLGN**	4.0	0.002
		**SC**	-0.09	0.92
		**SCN**	0.08	0.93
	**IBA-1 Perimeter**	**OT**	2.7	0.04
		**dLGN**	5.4	0.01
		**vLGN**	0.62	0.54
		**SC**	-0.31	0.75
		**SCN**	0.32	0.75
	**GFAP**	**OT**	-2.6	0.02
		**dLGN**	-6.4	<0.001
		**vLGN**	-5.3	<0.001
		**SC**	-4.9	<0.001
		**SCN**	-0.79	0.44

Supplementary Table S4. Student's t test results for all histology markers. If marked as "U=" a non-parametric Mann Whitney Rank Sum test was used. Abbreviations used correspond to regions spelled out in the first FJ-C section.

Supplementary Table S5. Endoplasmic Reticulum Stress (Western Blot) Statistical Results			
**Cohort**	**Region**	**t (or U)**	**p-value**
**Cohort 1**	**PERK**	U=26	0.006
	**IRE-1α**	-2.6	0.02
	**CHOP**	-5.7	<0.001
	**PDI**	0.81	0.42
	**ERO1L**	-2.4	0.02
	**Caspase**	-5.8	<0.001
**Cohort 2**	**PERK**	0.12	0.90
	**IRE-1α**	2.2	0.04
	**CHOP**	-2.5	0.03
	**PDI**	2.2	0.04
	**ERO1L**	-2.1	0.04
	**Caspase**	-0.3	0.7

Supplementary Table S5. Student's t test results for all immunoblotting proteins examined. If marked as "U=" a non-parametric Mann Whitney Rank Sum test was used.
